# An efficient high-gain bidirectional interleaved boost converter for PV integration to DC microgrid

**DOI:** 10.1371/journal.pone.0301522

**Published:** 2024-05-22

**Authors:** Edara Sreelatha, A. Pandian, Ch. Rami Reddy, M. Kiran Kumar, Hossam Kotb, Kareem M. AboRas, Abdulaziz Alkuhayli, Yazeed Yasin Ghadi, Ambe Harrison

**Affiliations:** 1 Department of Electrical and Electronics Engineering, Koneru Lakshmaiah Education Foundation, Guntur, India; 2 Department of Electrical and Electronics Engineering, Joginpally B R Engineering College, Hyderabad, India; 3 Applied Science Research Center, Applied Science Private University, Amman, Jordan; 4 Department of Electrical Power and Machines, Faculty of Engineering, Alexandria University, Alexandria, Egypt; 5 Electrical Engineering Department, College of Engineering, King Saud University, Riyadh, Saudi Arabia; 6 Department of Computer Science and Software Engineering, Al Ain University, Abu Dhabi, UAE; 7 Department of Electrical and Electronics Engineering, College of Technology (COT), University of Buea, Buea, Cameroon; J.C. Bose University of Science and Technology, YMCA, INDIA, INDIA

## Abstract

The design of a power electronic interface for high voltage difference DC buses is a key aspect in DC microgrid applications. A multi-port non isolated interleaved high-voltage gain bidirectional converter, which facilitates bidirectional power transfer and islanded operation in a DC microgrid, is presented in this paper. The forward high-voltage transfer ratio is achieved using a voltage multiplier circuit, and the high-gain step-down power conversion is performed using a resonant power module. A novel power transfer selection algorithm is proposed to control power flow among the interfaces of the RES, ESS, and DC grid converters, which utilizes the net power difference as the basis for switching the converter. The proposed converter is simulated for a 24 V PV source, 12 V battery, and 400 V DC grid interface using MATLAB/SIMULINK. A 200 W hardware prototype is implemented. The simulation results for voltages, currents, and power flow among RES, ESS, and microgrid DC bus proved an excellent voltage regulation, efficient power conversion, and a feasible duty cycle range with high voltage gain. These observations are validated through equivalent experimental results. A comparison is made regarding achieved gain, component sizing, achievable power transfer modes, efficiency, and control complexity with existing converters for DC microgrid applications. The presented topology proved to be a better interface with multiple-mode support with high efficiency.

## 1. Introduction

Renewable energy sources, such as photovoltaic (PV), have gained increased attention in response to the global energy crisis. However, the PV energy conversion system suffers from poor conversion efficiency due to multiple conversion stages and the high voltage difference between the source and load. To address these challenges, a high-gain step-up DC-DC converter is employed to elevate the low-voltage profile of photovoltaics. This section provides the requirements, topologies, control, and drawbacks of existing power converters for high-gain DC-DC conversion. High-gain DC-DC conversion is required for various applications, including automobile power systems, DC microgrids, multi-port conversion, traction, and renewable integration [1, 2]. However, the intermittent nature of natural sources requires a secondary energy storage system [3, 4] with an energy management system to provide a quality uninterruptible power supply. It is achieved by hybrid renewable energy systems (HRES) [5]. HRES has become more popular, used and implemented worldwide for reliable and quality power generation [6, 7]. The DC grid is preferred for its good power quality, efficiency, and performance compared to distribution systems [1, 8]. This also provides high grain output with a transformer-less setup [9, 10]. The boost converter’s lower efficiency and higher duty ratio operation limit the use in high-gain conversion. Moreover, the bulky inductor for continuous current conduction and reverse recovery of the power switch also becomes a central issue in high gain conversion [5, 11]. To overcome the issues, cascaded boost converters were launched. It produces high-gain output at a low-duty cycle. However, it is affected by poor efficiency, stability, and complex control techniques [1]. So, integrated DC-DC boost converters with more than one phase were proposed. In the interleaved converter topology, the increase in phases reduces input current ripple content. To further increase the gain, voltage multiplier circuits (VMC) such as Dickson VMC and Cockcroft-Walton VMC are used [12]. The switched capacitor and the switched inductor converter [13, 14] provide a very high step-down conversion. It is used to supply low-voltage applications such as electric vehicles (EVs) [15] and the low-voltage energy storage system used in HRES [16]. The low-voltage nature of the sources and medium/high-voltage DC distribution system [17, 18] need to gain power converter topologies. A transformer obtains a high gain in the isolated DC-DC converter topologies. High-frequency transformers provide sufficient gain and isolation between the input and output ports. The use of high-frequency transformers and multistage power converters increases cost, size, weight, losses, and low efficiency [19]. To overcome the drawbacks mentioned above, non-isolated DC-DC converter circuits are preferred [20, 21].

Cascaded boost stages [22, 23], quasi-Y sources [24], switched coupled inductors [25] are developed to achieve high voltage gain with interleaved boost converters. Leakage inductance stresses switches in the DC-DC flyback converter, which has a simple construction and high voltage gain [26]. Energy-regeneration strategies have been applied to mitigate this stress. On the other hand, the phase-shifted full-bridge converter generates higher input ripple currents. It achieves a high increase gain by using a higher number of turns ratio in the transformer [27, 28]. Electrolytic capacitors are used in the phase-shifted full-bridge converter to reduce input current ripples. Further opportunities to achieve higher efficiencies include soft switching and low-duty cycle operation of the converter. Switching capacitor-based converter circuits [29, 30], such as active-clamp dual boost and complete bridge boost converters, provide zero voltage and zero current switching to obtain reduced switching losses. However, these converters have recently experienced a high transient loss, significant switch conduction loss, and increasing complexity [31] with additional switched capacitor cells. Modified switched capacitor cells use soft switching [32] to decrease switching loss and electromagnetic interference. A coupled inductor approach with a modified turn ratio [33] also significantly increases gain. However, all currently used high step-up ratio and ultra-step-up converters still experience significant voltage stress [34] across the diode. Other nonconventional unidirectional converters include dual active bridges for microgrids [35], transformer-less ultra-high gain conversion [36, 37], and multi-port conversion [38, 39]. Advances in current regulation include voltage stabilization [40, 41], state observer for battery current control [12, 42], and the model-based current reconstruction method [43]. The decentralized DC microgrid voltage control structures were reported in [6, 44]. Optimal control for energy storage charge / discharge methods [45, 46], artificial intelligence such as fuzzy inference [47], ANN-based island control [48, 49] were developed to find the reliability study for microgrids [50].

Thus, it is observed that the existing converters are either bulky or independent stages for multi-port power conversion. Therefore, there is a need to develop an interface that achieves high gain with a feasible duty cycle and higher efficiency in multi-port power conversion.

The novel contributions of the proposed converter and control include,

- Achieving Bi-directional and islanded mode power transfer in DC microgrid applications.
- Achieving high gain in both forward and reverse-power transfer mode.
- Design of converter interface that provides multi-port operation for integrating two sources to DC microgrid along with interleaved operation on the availability of either of the sources.
- Uninterrupted power conversion between three ports with automatic power transfer mode selection algorithm.
- Configurable multiplier to obtain high gain at a feasible duty cycle of converter switches.

The rest of the paper is organized as follows—the schematic of the multi-port DC-DC converter discussed in Section 2. The working principle of the proposed converter circuit in each power transfer mode, control structure, and algorithm are discussed in Section 3. The MATLAB simulation analysis, the experimental setup and its results, and the performance comparisons are explained in Section 4. The conclusion and future work are discussed in Section 5.

## 2. Multiport dc-dc converter for grid integration

**Fig 1:**
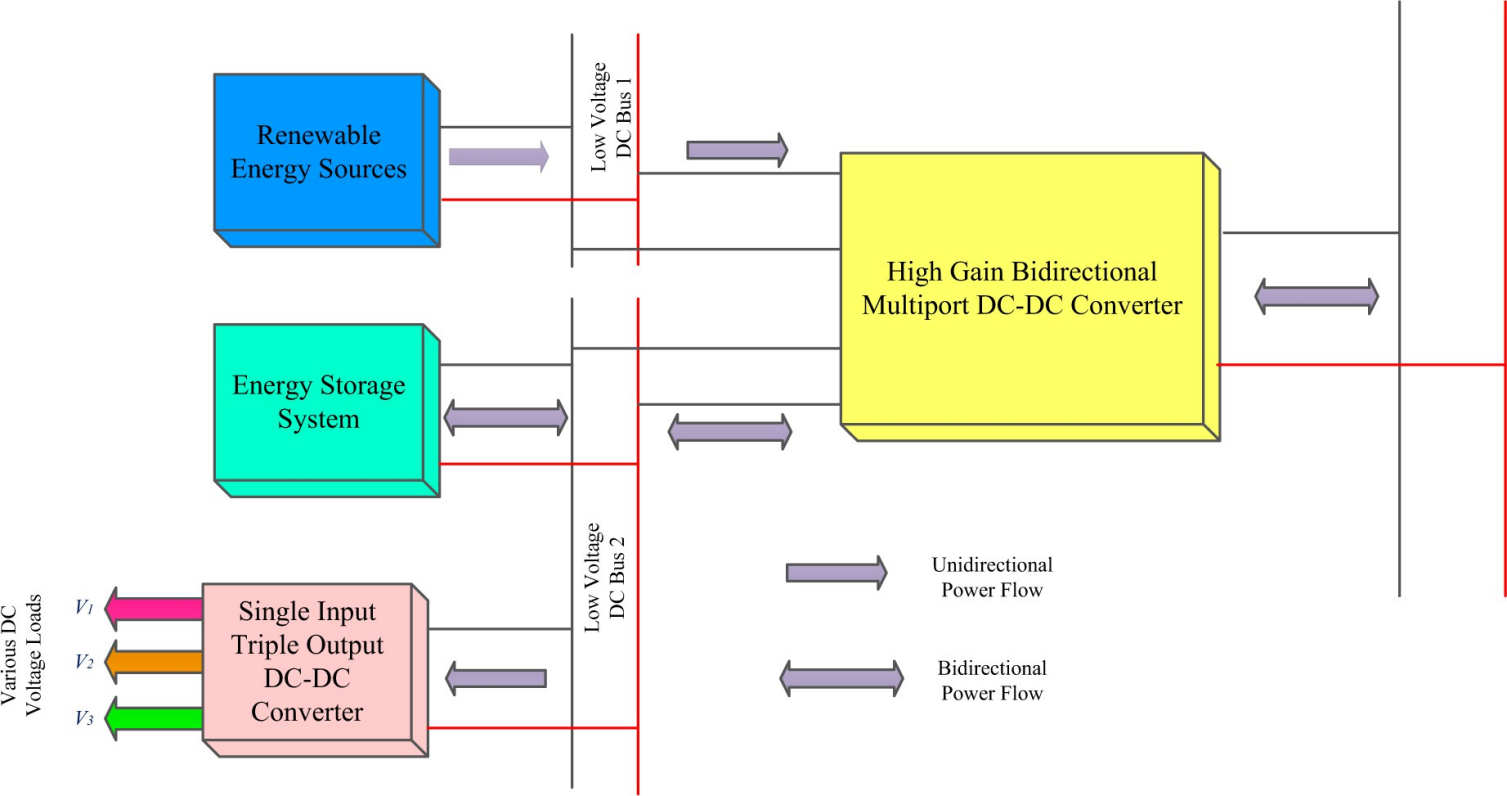
Block diagram of the multi-port DC-DC converter.

**Fig 2:**
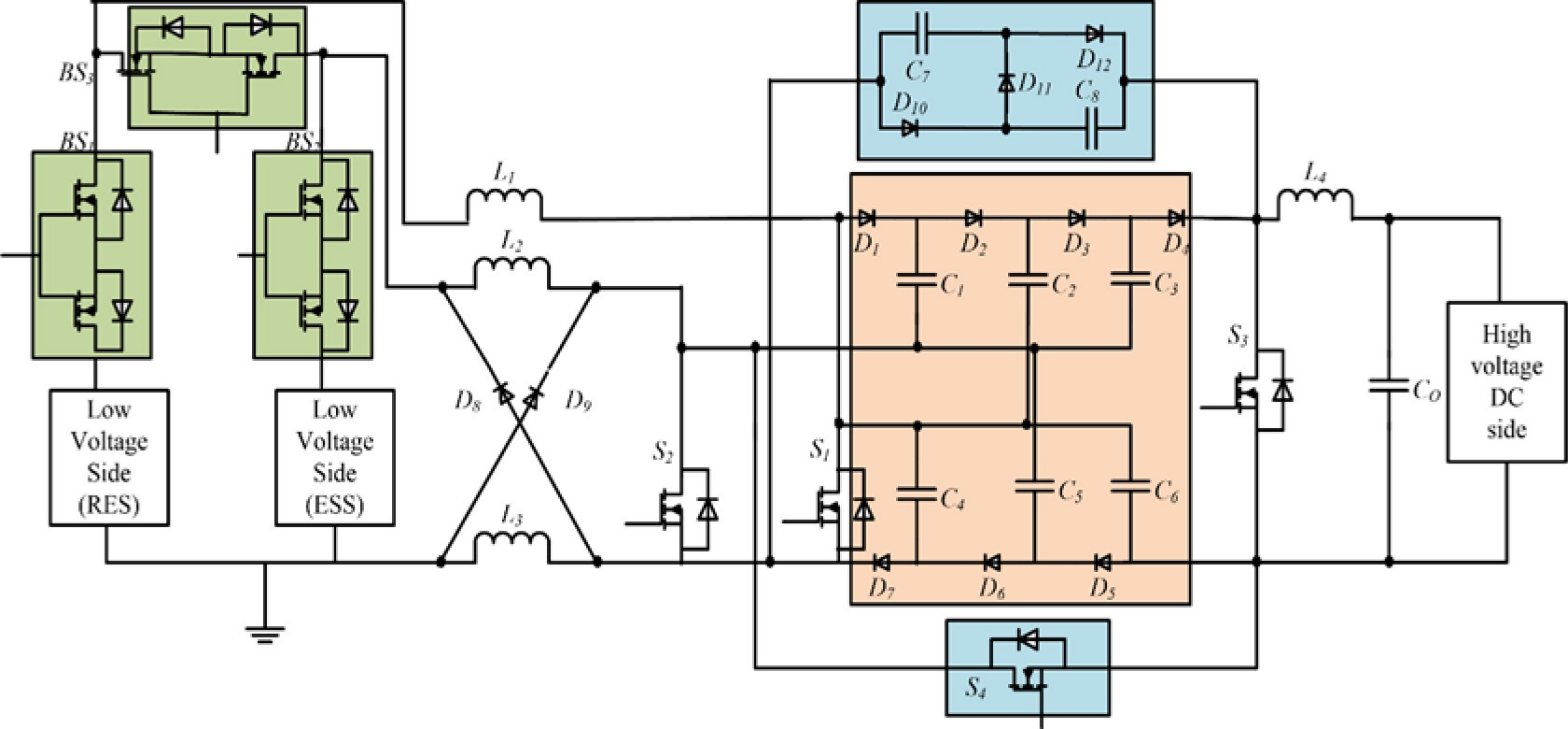
Interleaved boost converter circuit with three-level VMC and SCSL module.

The bidirectional multi-port DC-DC converter block diagram is shown in Fig 1, and the proposed circuit is shown in Fig 2. The circuit is made up of four unidirectional controllable power semiconductor switches and three bidirectional controllable power semiconductor switches with diodes and energy storage elements capacitors and inductors. High-gain three levels of VMC are used with a front-end interleaved converter, and to get high-gain step-down conversion, a switched capacitor and switched inductor module are used. The construction and operation of the proposed topology are explained separately.

## 3. High-gain bidirectional multi-port dc-dc converter

This section provides a detailed description of the operation of the converter under various modes of power transfer among the available renewable energy source (RES), energy storage system (ESS), and DC grid interfaces of the proposed converter. The topology of the converter, the equivalent circuit of the ON and OFF states in each mode, the mathematical analysis and the control structure are presented as follows.

### 3.1 Constructional parts of the proposed converter circuit

#### 3.1.1 Interleaved boost stage

**Fig 3:**
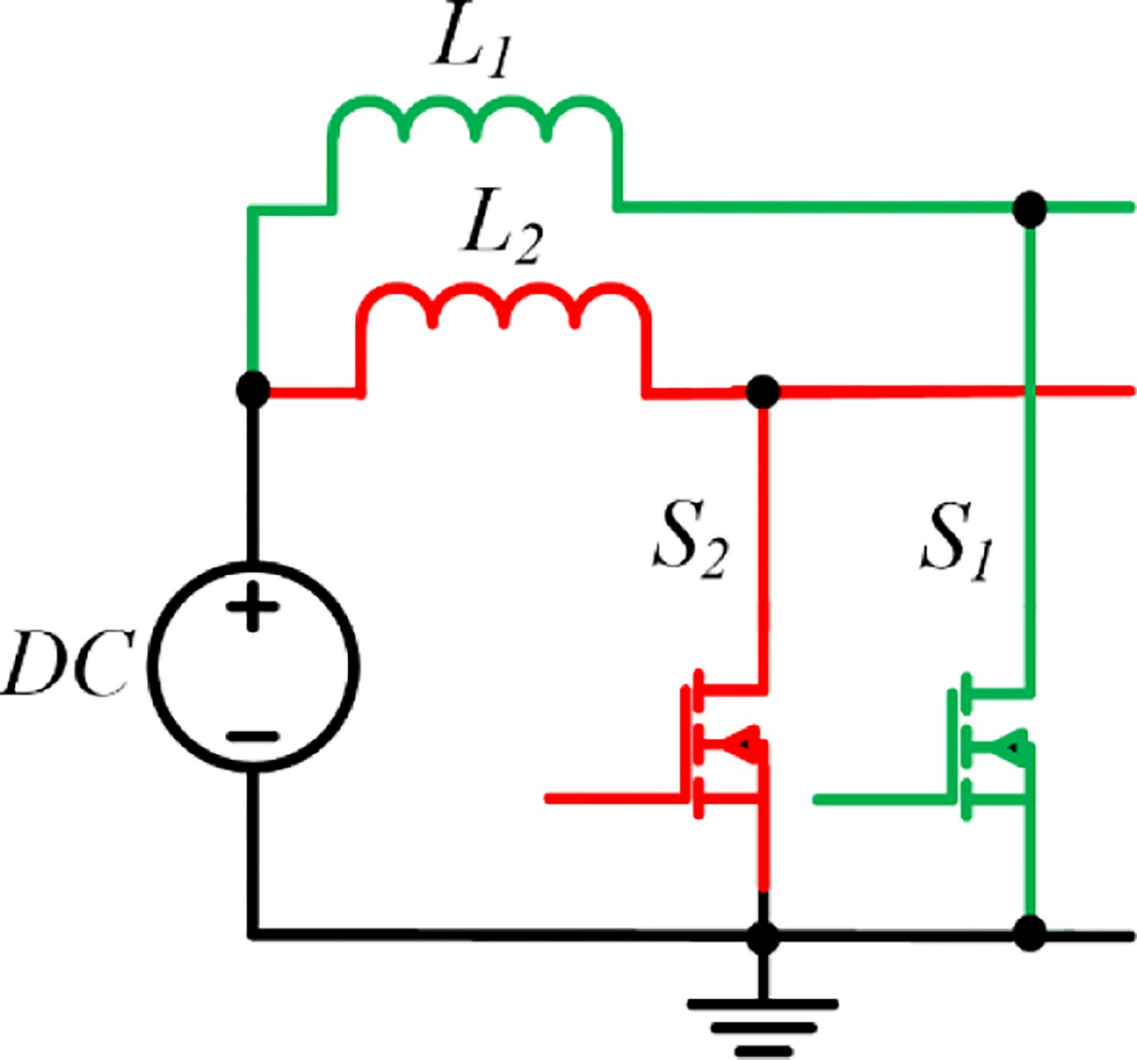
The two-phase interleaved boost stage.

As shown in Fig 3, the interleaved boost stage comprises an inductor and a power switch as a conventional boost converter. However, each stage has a phase difference to minimize the input ripple current.

**Fig 4:**
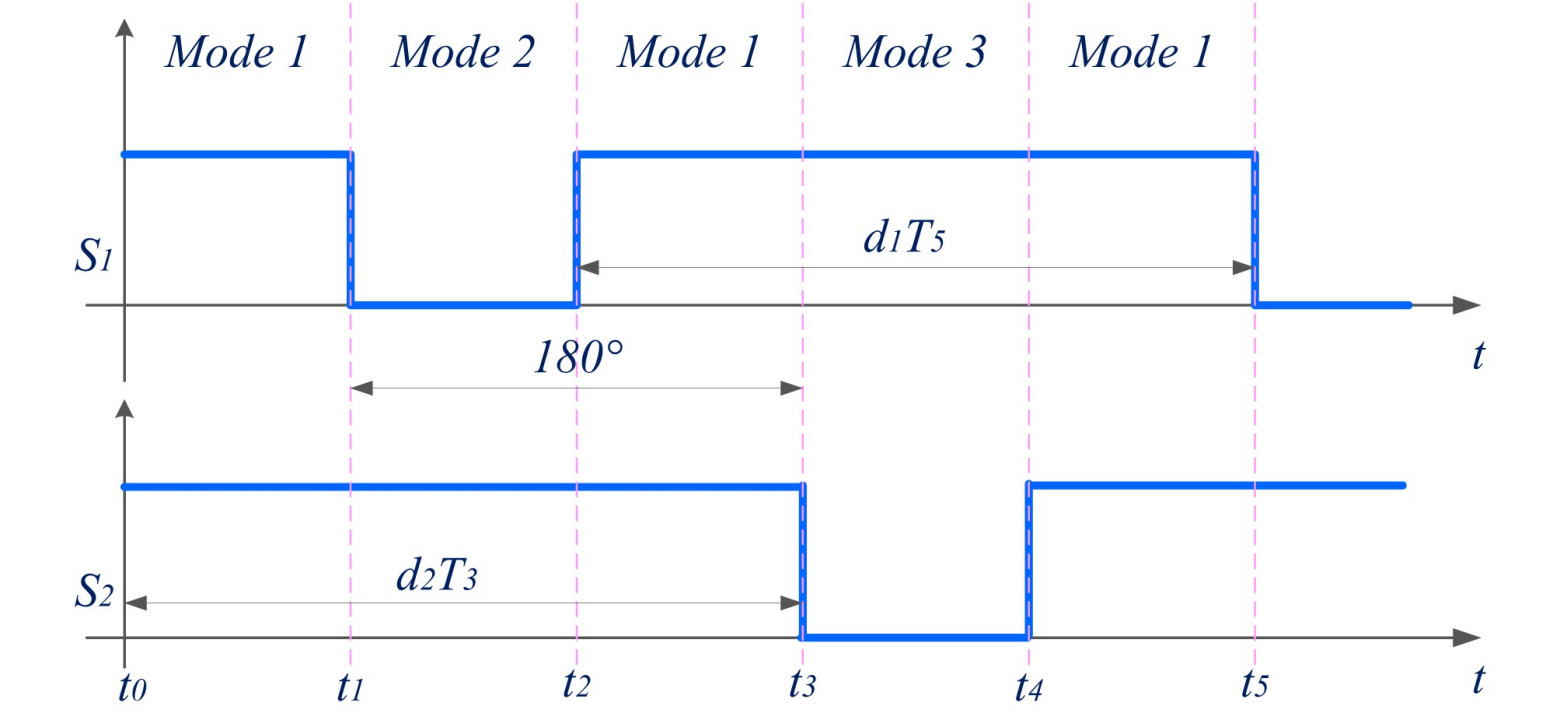
Two-phase interleaved boost stage: Switching pattern.

The phase change among stages is determined by the number of stages presented: an increase in phases reduces the input ripple current. However, the maximum number of stages is limited by cost, size, weight, and control complexity. As it is a current source, there is no need to provide dead time. In the proposed converter topology, two-phase interleaved boost stages are used. The switching pattern is shown below in Fig 4.

#### 3.1.2 Voltage multiplier stage

**Fig 5:**
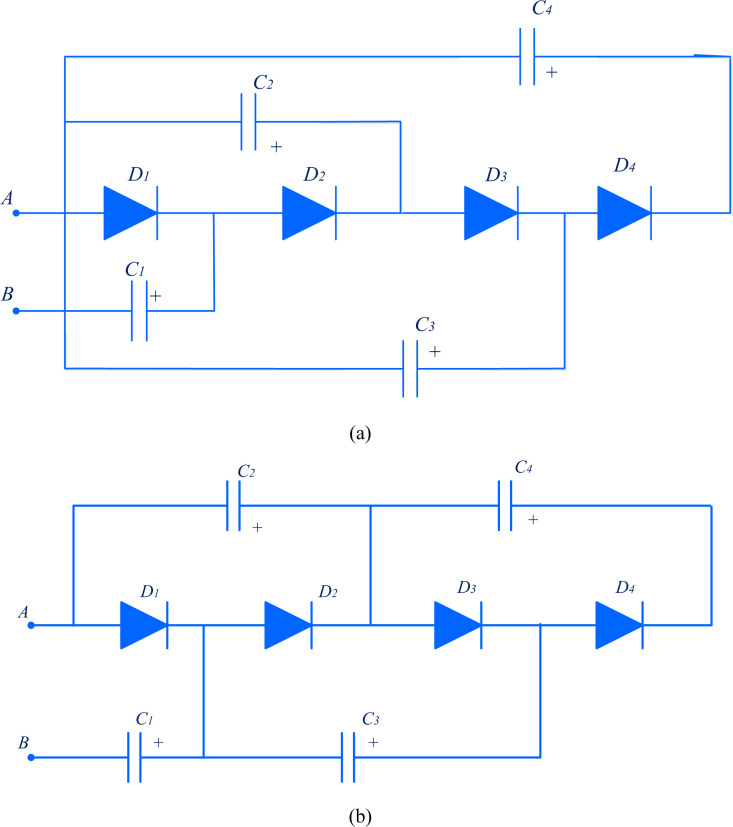
Voltage multiplier circuit (a) Dickson VMC, (b) Cockcroft- Walton VMC.

Capacitors are utilized to increase the input voltage. The diodes are connected in such a way as to charge the capacitors to get more voltage. The number of voltage multiplier stages calculates the magnitude of voltage step-up. The amount of power transfer is based on the size of the capacitors. Dickson VMC and Cockcroft-Walton VMC are shown in Fig 5(A) and 5(B).

Both work with AC input voltage. The uncontrolled nature of power diodes does not require a control signal and gate driver circuits. It eliminates control and driver circuits. However, interleaved boost converters at the front end convert the available DC power supply into high-frequency AC

#### 3.1.3 Switched capacitor switched inductor (SCSL) module

**Fig 6:**
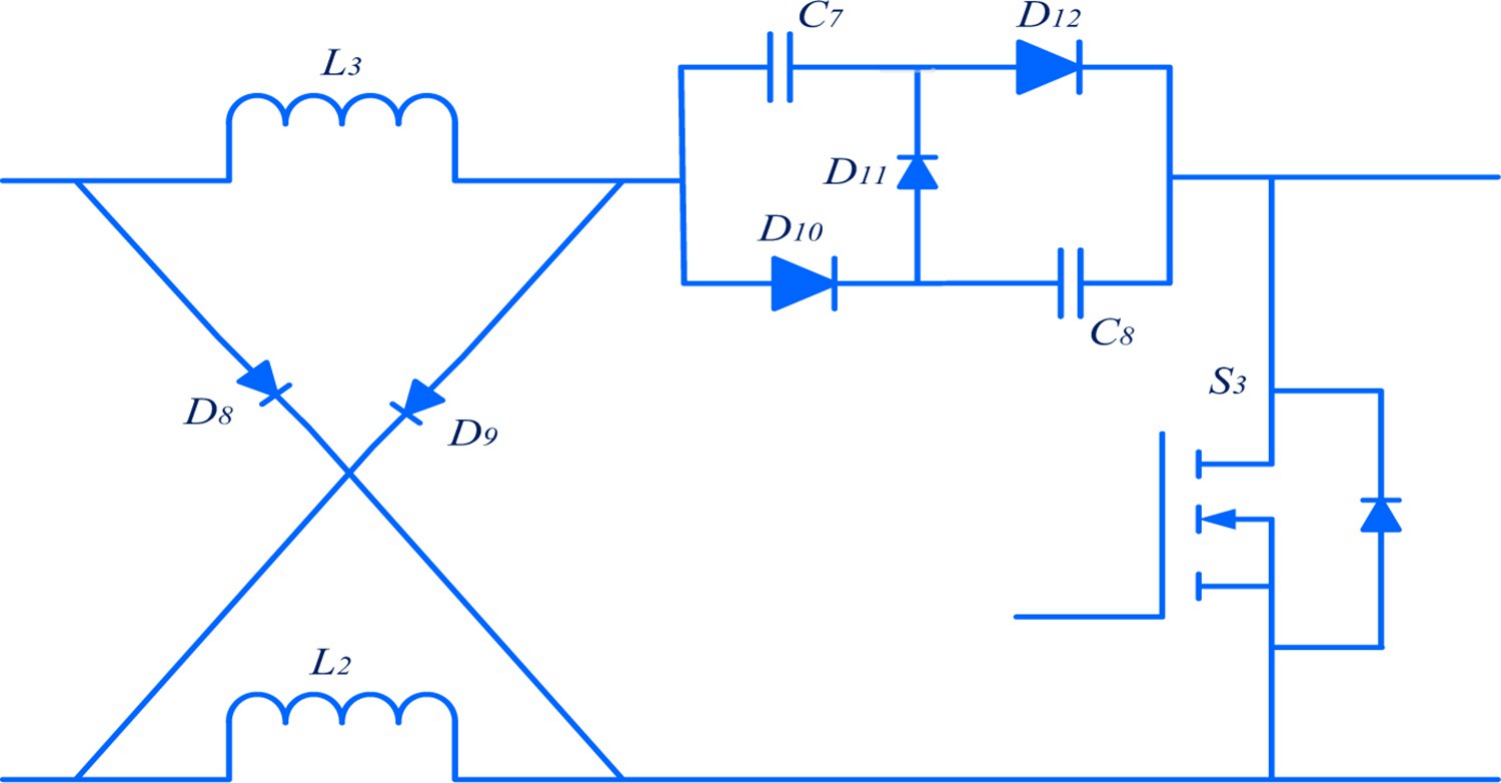
Cuk-derived switched capacitor and switched inductor.

SCSL obtains the reverse high voltage transfer ratio shown in Fig 6, which is derived from the topology of the cuk converter. This module acts as the interface to transfer the power from the high-voltage DC side to the low-voltage DC side with high efficiency.

### 3.2 Working of the proposed circuit

**Fig 7:**
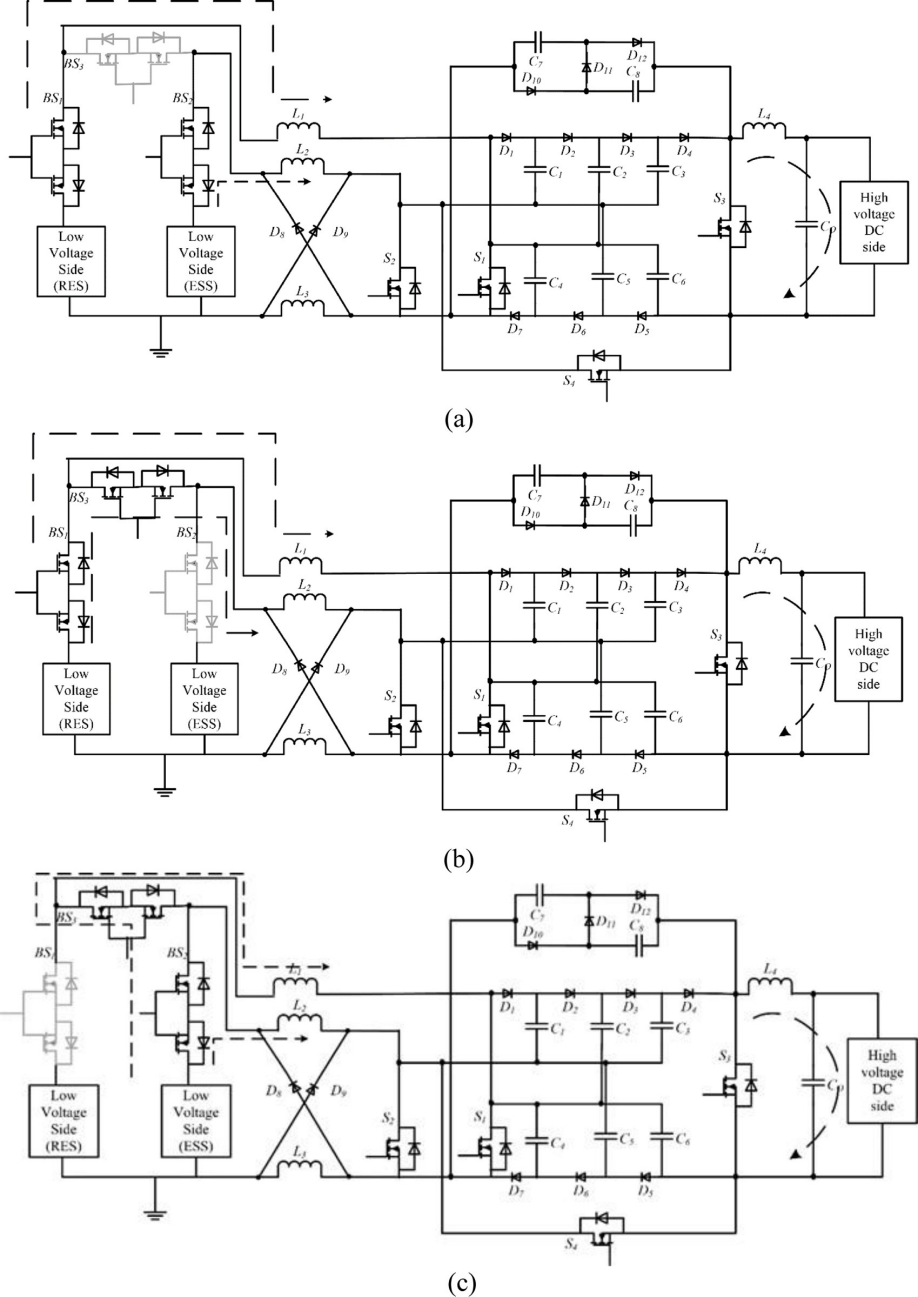
Interleaved operation of the proposed converter, (a) Interleaved operation using both sources, (b) Interleaved operation using a renewable energy source, and (c) Interleaved operation using the energy storage system.

The power transfer modes of the proposed topology are explained below. The interleaved boost operation of the converter to perform power transfer between the low-voltage and high-voltage sides is shown in Fig 7. Both legs must be operated with phase differences to perform the interleaved operation. One leg is connected to a renewable energy source and the other is connected to the battery energy storage system. The availability of each source is subject to change. So a source selection circuit is used to use the available source. The source selection circuit comprises three bidirectional switches, *BS*_*1*_, *BS*_*2*,_ and *BS*_*3*,_ shown in Fig 7.

#### 3.2.1 Interleaved boost operation

If both sources are available, the bidirectional switches *BS*_*1*_ and *BS*_*2*_ are activated and *BS*_*3*_ is disabled. Both sources are involved in the interleaved operation and deliver power to the high-voltage side, as shown in Fig 7(A). The bidirectional switches *BS*_*1*_ and *BS*_*3*_ are turned on and *BS*_*2*_ is turned off when no power is available in the energy storage system. The power is transferred from renewable energy sources to the load, as shown in Fig 7(B). Similarly, bidirectional switches, *BS*_*2*_ and *BS*_*3*,_ are turned on, and *BS*_*1*_ is turned off when no power is available in renewable sources. So, the total power is consumed from the energy storage systems, as shown in Fig 7(C).

#### 3.2.2 Step-up power transfer

In the step-up power transfer mode, the power transfer is performed from the low-voltage side to the high-voltage side, as shown in Fig 7. To perform this power conversion, the interleaved boost stage converters made up of switches *S*_*1*_ and *S*_*2*_ operated to charge the VMC. Then, the three-level VMC steps up the magnitude further, which is equal to the high-voltage DC distribution system. During this mode, control signals to other power switches are disabled. The operating modes are divided into three based on the switching states during step-up power transfer. They are

*a*. *Mode 1*. If both sources are available during this mode, the inductors *L*_*1*_ and *L*_*2*_ *are* charged by turning on switches *S*_*1*_ and *S*_*2*_. All of the diodes are under reverse bias in this mode.


$$v_{L_{1}}=v_{RES}$$ (1)

$$Which\;implies\;L_{1}\frac{di_{L1}}{dt}=v_{RES}$$ (2)

$$Then\;2L_{1}\;f_{sw}\frac{\Delta i_{L1}}{D}=v_{RES}$$ (3)

$$Similarly,\;v_{L_{2}}=v_{ESS}$$ (4)

$$L_{2}\frac{di_{L2}}{dt}=v_{ESS}$$ (5)

$$2L_{2}\;f_{sw}\frac{\Delta i_{L2}}{D}=v_{ESS}$$ (6)

If RES is alone and no ESS is available, then RES is selected to perform the interleaved operation using bidirectional switches. Then

$$v_{L_{1}}=v_{L_{2}}=v_{RES}$$ (7)

$$Then,\;{2L}_{1}\;f_{sw}\frac{\Delta i_{L1}}{D}=2L_{2}\;f_{sw}\frac{\Delta i_{L2}}{D}=v_{RES}$$ (8)

Similarly, if ESS is alone and no RES is available, EES is selected to perform the interleaved operation using the bidirectional switches. Then

$$v_{L_{1}}=v_{L_{2}}=v_{EES}$$ (9)

$$Then,\;2L_{1}\;f_{sw}\frac{\Delta i_{L1}}{D}={2L}_{2}\;f_{sw}\frac{\Delta i_{L2}}{D}=v_{ESS}$$ (10)

*b*. *Mode 2*. Switch *S*_*1*_ is kept in the ON state during this mode, but *S*_*2*_ is turned off. The inductor *L*_*1*_ stores energy continuously and *L*_*2*_ starts to discharge to the capacitors present in the voltage multiplier cell.

$$v_{L_{1}}=v_{RES}$$ (11)

$$Then,\;2L_{1}\;f_{sw}\frac{\Delta i_{L1}}{D}=v_{RES}$$ (12)

and

$$v_{L_{2}}=v_{ESS}-v_{c_{4}}$$ (13)

$${Then,\;2L}_{2}\;f_{sw}\frac{\Delta i_{L2}}{D}=v_{ESS}-v_{c_{4}}$$ (14)

*c*. *Mode 3*. Switch *S*_*1*_ is turned off and *S*_*2*_ is turned on during this mode. Inductor *L*_*1*_ discharges stored energy towards the capacitors in the voltage multiplier cell.


$$v_{L_{1}}=v_{RES}-v_{c_{1}}$$ (15)

$$Then,\;2L_{1}\;f_{sw}\frac{\Delta i_{L1}}{D}=v_{RES}-v_{c_{1}}$$ (16)

$$v_{L_{2}}=v_{ESS}$$ (17)

$${Then,\;2L}_{2}\;f_{sw}\frac{\Delta i_{L2}}{D}=v_{ESS}$$ (18)

The steady-state voltage gain is now obtained as the volt-sec balance across each inductor pertaining to *Δi*_*L*1_ = 0, and *Δi*_*L*2_ = 0 which shall be obtained if

$$\langle v_{L_{1}}DT+v_{L_{1}}(1-D)T\rangle =0\;and\;\langle v_{L_{2}}DT+v_{L_{2}}(1-D)T\rangle =0$$ (19)

$$This\;provides,\;v_{c_{1}}=\frac{V_{RES}}{1-D}$$ (20)

$$And,\;v_{c_{4}}=\frac{V_{ESS}}{1-D}$$ (21)

The voltage across the capacitors,

$$\nu_{C_{1}}=\frac{v_{RES}}{1-D}$$ (22)

$$v_{C_{2}}=\frac{2v_{RES}}{1-D}$$ (23)

$$v_{C_{3}}=\frac{3v_{RES}}{1-D}$$ (24)

$$\nu_{C_{4}}=\frac{v_{ESS}}{1-D}$$ (25)

$$v_{C_{5}}=\frac{2v_{ESS}}{1-D}$$ (26)

$$v_{C_{6}}=\frac{3v_{ESS}}{1-D}$$ (27)

$$V_{0}=\frac{3}{1-D}v_{RES}+\frac{3}{1-D}v_{ESS}$$ (28)

$$M=\frac{2*v_{0}}{{(v}_{RES+v_{ESS}})}=\frac{7}{1-D}$$ (29)

So, the transfer function of the step-up model is

$$M=\frac{2N+1}{1-D}$$ (30)

where *N* is the number of stages in the VMC

#### 3.2.3 Power transfer between two low-voltage sources

**Fig 8:**
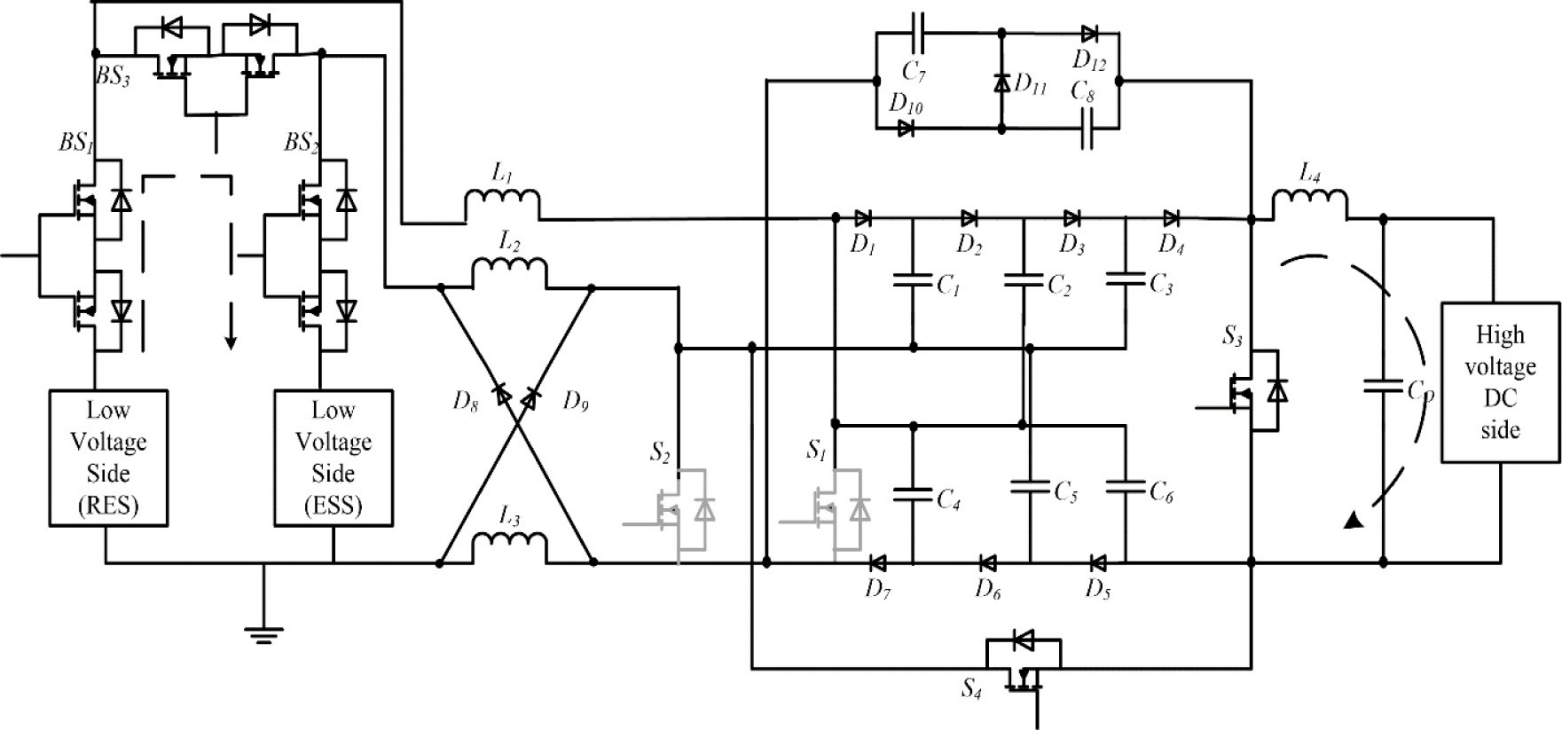
Power transfer from RES to ESS.

In the two input sources, one is the RES and the other is the battery. The battery is charged using a bidirectional controllable switch *BS*_*3*,_ as shown in Fig 8. The battery is charged when excess power is found from renewable sources. This mode of operation is independent of step-up power transfer between low-voltage sides to the DC distribution system.

The power transfer is done by step-down conversion like a basic converter; the transfer function in this mode,

$$\frac{v_{0}}{v_{RES}}=D$$ (31)

#### 3.2.4 Step-down power transfer

**Fig 9:**
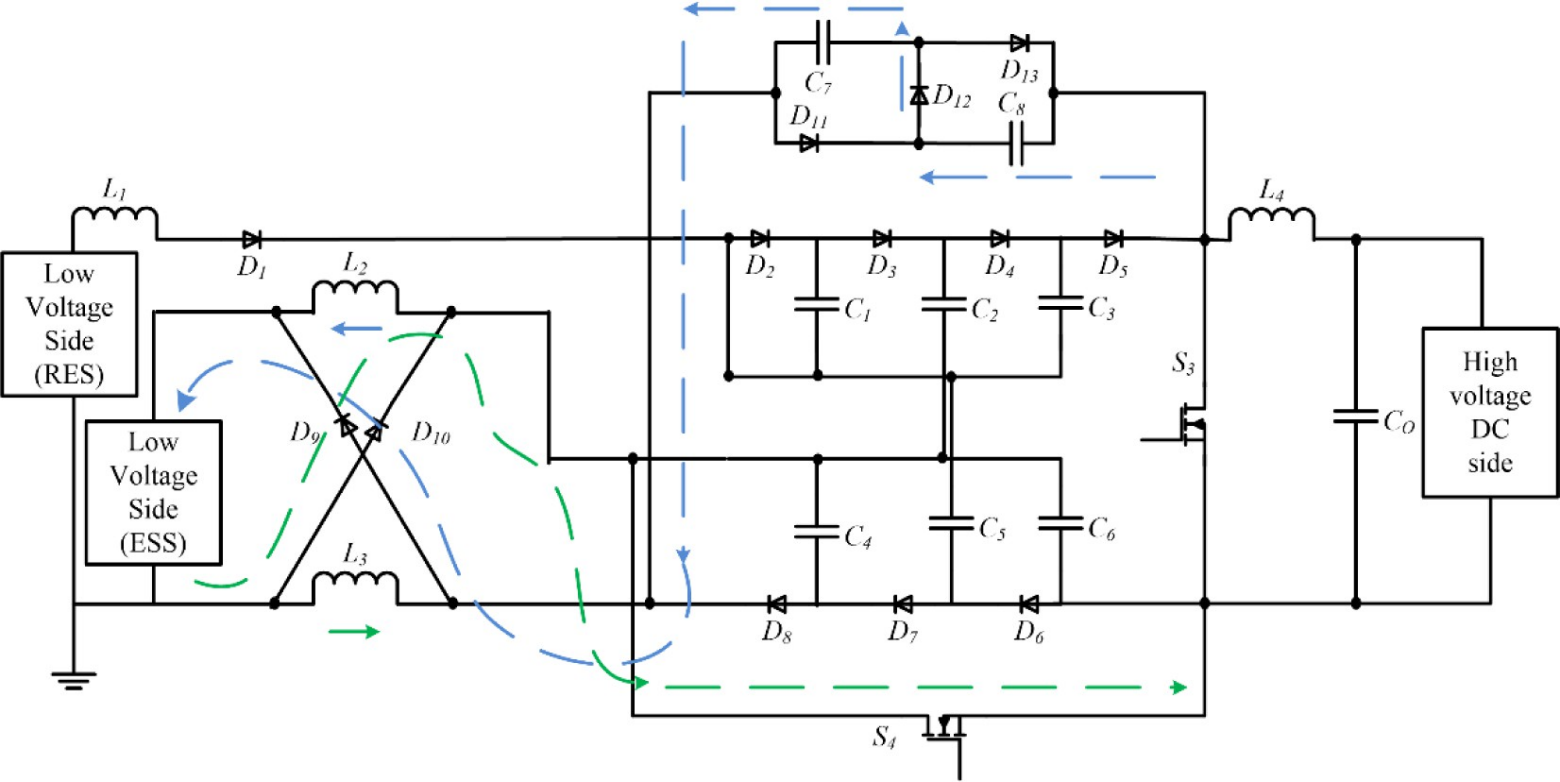
High-voltage side to low-voltage DC side power transfer.

The power transfer from the DC distribution to the battery is done through the SC-SL module. First, the capacitor is charged from the output voltage and then the initially discharged inductors are charged from the capacitors. Then the energy stored in the SC-SL is transferred to the low-voltage DC side, as shown in Fig 9. During the operation, the switching pulses to the interleaved boost stage are disabled.

The transfer function during the drop-down mode as obtained from [34] is

$$\frac{v_{0}}{v_{HV}}=\frac{D}{\left(4-D)(1-D\right)}$$ (32)

### 3.3 Power transfer mode selection control algorithm

**Fig 10:**
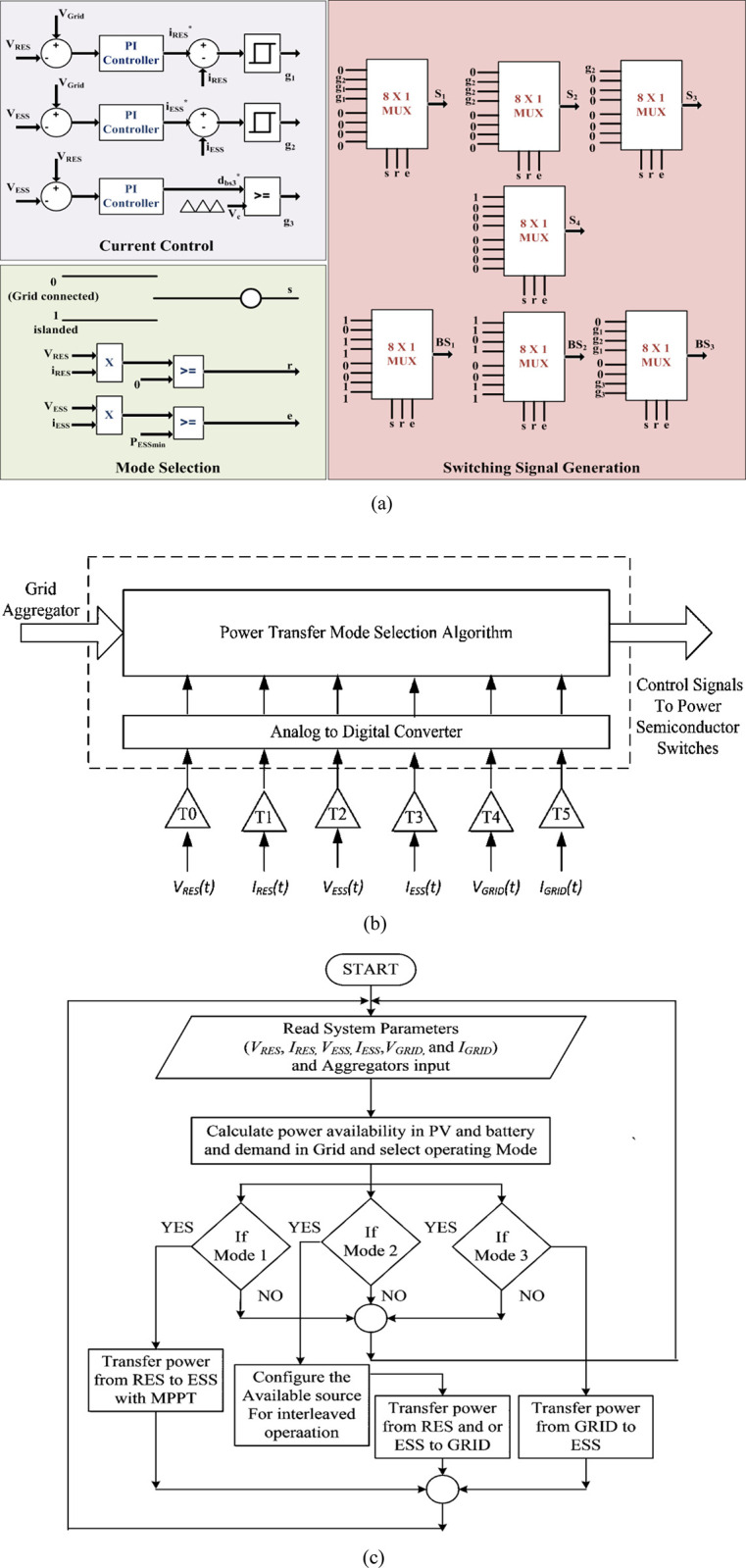
(a) Control structure; (b) Block diagram of power transfer mode control algorithm; (c) Flow chart for mode selection.

The detailed control structure for automatic mode selection, current regulation, and gating signal generation is presented in Fig 10(A). The measured voltages and currents at the respective terminals calculate the power at the RES and ESS terminals. Positive RES power at a minimum terminal voltage and ESS power greater than critical value determines the mode for supplying energy to the DC microgrid or charging the battery. The manual grid connection command, along with RES and ESS power availability, thus forms five modes of power transfer as depicted in Table 1, which generate three-bit binary control lines of 8 x 1 mux to route pwm signals to respective switches. Current regulation at RES and ESS terminals follows the hysteresis current control with reference generated from the PI. Controller current commands are based upon the voltage difference of the respective bus with microgrid DC bus. The voltage difference between RES and ESS buses determines the duty cycle for power transfer between RES and ESS. The gating signal is generated accordingly compared to the constant frequency carrier. Finally, the gating signals are generated for all switches through 8 X 1 mux with control lines as mode selection signals. The respective PWM pulses are routed to the switches according to the mode determined, as shown in Fig 10(A).

**Table 1:** Power transfer modes.

Operating Mode		Renewable Energy	Stored Energy	Power transfer details
Mode 1	Grid Connected Mode	Available	Available	Power transferred from RES and ESS to Grid side
Mode 2		Available	Not available	Power transferred from RES to Grid side
Mode 3		Not available	Available	Power transferred from ESS to Grid side
Mode 5		Not available	Not available	Power transferred from Grid to ESS
Mode 4	Islanded Mode	Available	Not available	Power transferred from RES to ESS

The detailed mode selection algorithm is shown in Fig 10(B). In the power transfer mode selection control algorithm, the system parameters are measured instantly and conditioned through a voltage divider circuit with respective gains T0 –T5 converted into equivalent digital form. It is supplied for the control algorithm as input. The control signals are generated based on the input of the grid aggregator. The aggregator provides uninterrupted grid balancing to optimize energy use and pays its customers for making their electricity available. The different power transfer modes are listed in Table 1, and the flow chart representation is shown in Fig 10(C). The available power in RES and ESS is measured and compared to the power demand in the DC grid. The sign and magnitude of the power difference at the DC grid and the ESS interface determine the extension of the gating signals from *S*_*1*_ to *S*_*3*_. Then, the duty cycle is selected from the magnitude of power to be supplied to each interface.

## 4. Results and discussion

The proposed converter and power control algorithms were tested for steady-state power transfer through a simulation study and the same was validated using a hardware prototype. This section presents the simulation results for voltages and currents at each converter interface.

### 4.1 Simulation results

**Fig 11:**
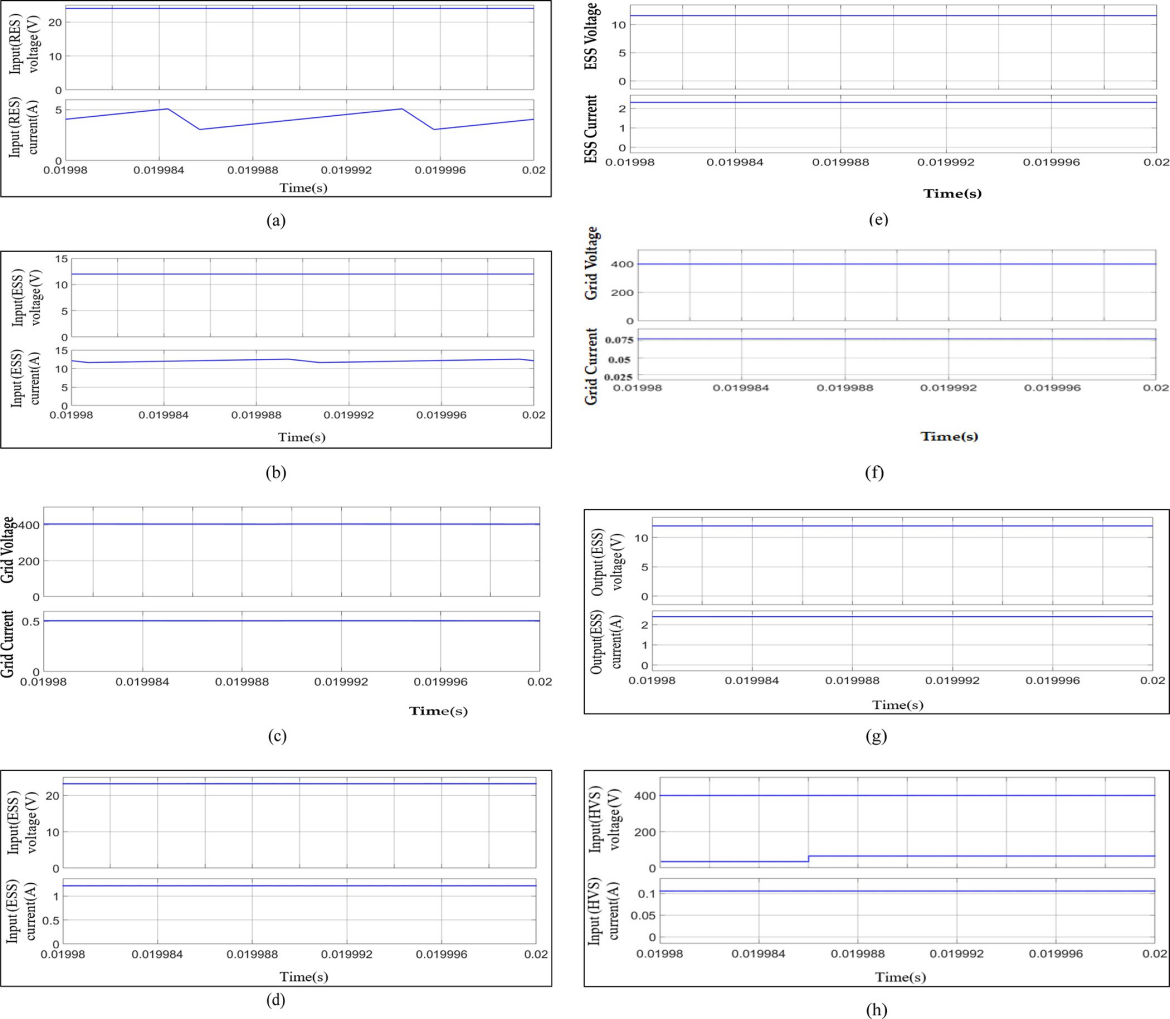
Simulation waveforms for (a) Voltage and current waveforms at RES in Mode 2, (b) Voltage and current waveforms at ESS in Mode 2, (c) Voltage and current waveforms at the DC grid in Mode 2, (d) Voltage and current waveforms at RES in Mode 1, (e) Voltage and current waveforms of ESS in Mode 1, (f) Voltage and current waveforms at the DC grid in Mode 3, (g) Voltage and current waveforms at ESS in Mode 3, (h) DC grid voltage for varying voltage at RES.

The proposed converter circuit is simulated using the MATLAB computer simulation tool Matlab. The maximum step size and tolerance are 10-9s and 10–3, respectively. The ode23tb solver is chosen to simulate the circuit. The simulation parameters are listed in Table 2, and the steady-state waveforms for voltages and currents at various interfacing nodes are plotted in Fig 11(A)–11(G).

**Table 2:** Simulation parameters.

Parameter	Value
RES	24 V
ESS	12V, 10 AH
DC grid Voltage	400 V
Load resistance at the DC grid	800 Ω
Duty cycle	0:65
Maximum Switching frequency	100 kHz
Hysteresis Band	1 percent
*L*_*1*_ *–L*_*4*_	100 μH
*C*_*1*_ *–C*_*8*_	10 μF

The magnitudes of steady-state voltages and currents at the RES interface, the ESS interface, and the DC grid interface of the converter are shown in Fig 11(A)–11(C), respectively. This condition represents mode two operation in Section 3, with RES and ESS supplying the required power to the DC grid. The total power demand in the DC grid is 200 W, of which 144 W was from ESS and 60 W from RES. The magnitudes of steady-state voltages and currents at the RES interface and ESS interface of the converter are shown in Fig 11(D) and 11(E), respectively, for mode one operation about no power demand at the grid and ESS with shortage of threshold power. The ESS is charging at 12 V with a power of 28.8 W. The total power supplied by RES at 24 V is shown in Fig 11(D). The magnitudes of steady-state voltages and currents at the DC grid interface and the ESS interface of the converter are shown in Fig 11(F) and 11(G), respectively, for mode three operation about the unavailability of RES and ESS with shortage of threshold power. The ESS is charging at 12 V with a power of 28.8 W. The required, as observed from Fig 11(F), is supplied by the DC grid at 400 V. Fig 11(H) presents the DC grid voltage regulation for the change in RES voltage from 24 V– 30 V, which shows a negligible change in the DC grid voltage, proving the robustness of instantaneous duty cycle control for changing input.

The size of the interleaved inductors for the desired maximum ripple current is obtained as follows.

From Eq (3),

$$L_{1}=\frac{D}{{2f}_{sw}\Delta i_{L1}}v_{RES}$$ (33)

Also, from Eq (29) under RES operating alone,

$$D=1-\frac{7*V_{RES}}{V_{Grid}}$$ (34)

Now, to determine the worst-case duty cycle, consider the range of RES voltage varying from 22 V– 26 V in the operating range of irradiance, duty cycle variants from 0.545–0.615. Substituting the maximum value of the operating duty cycle, the size of the inductor L_1_ can be determined as

$$L_{1}=\frac{0.615*22}{{2f}_{sw}\Delta i_{L1}}$$ (35)

From Eq (6),

$$L_{2}=\frac{D}{{2f}_{sw}\Delta i_{L2}}v_{ESS}$$ (36)

Also, from Eq (29) under ESS operating alone,

$$D=1-\frac{7*V_{ESS}}{V_{Grid}}$$ (37)

Now, to analyze the worst-case duty cycle, consider the range of ESS voltage varying from 10.2 V to 13.8 V in the operating range of irradiance, duty cycle variations from 0.7585 to 0.8215. Substituting the maximum value of the operating duty cycle, the size of the inductor *L*_*2*_ can be determined as

$$L_{2}=\frac{0.8215*10.2}{{2f}_{sw}\Delta i_{L2}}$$ (38)

These were verified from Fig 11(A) and 11(B) with the observed ripple in RES and ESS currents to be 0.01 pu for the designed inductor sizes as depicted in Table 2.

### 4.2 Experimental results

**Fig 12:**
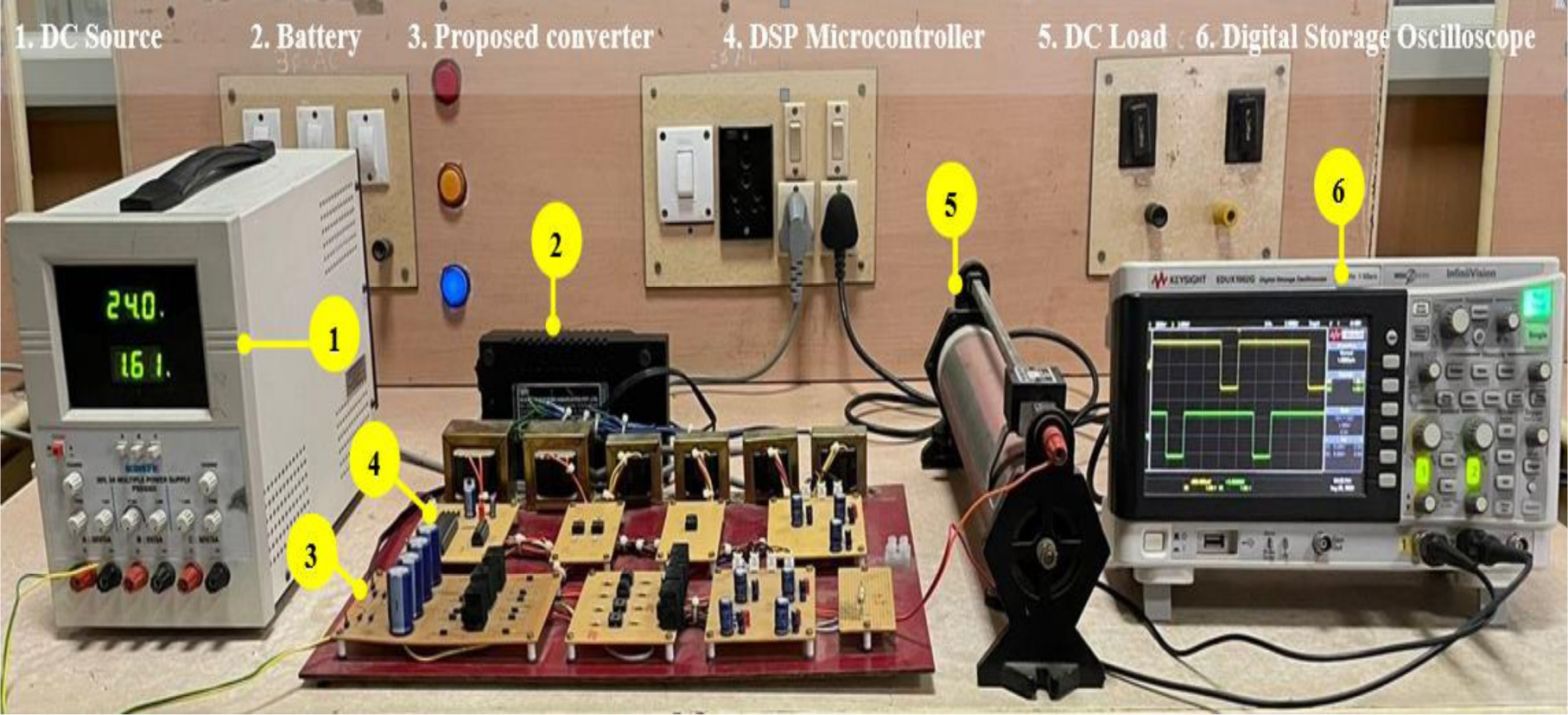
Hardware prototype and experimental setup of an interleaved boost converter with three-level VMC and SCSL module.

**Fig 13:**
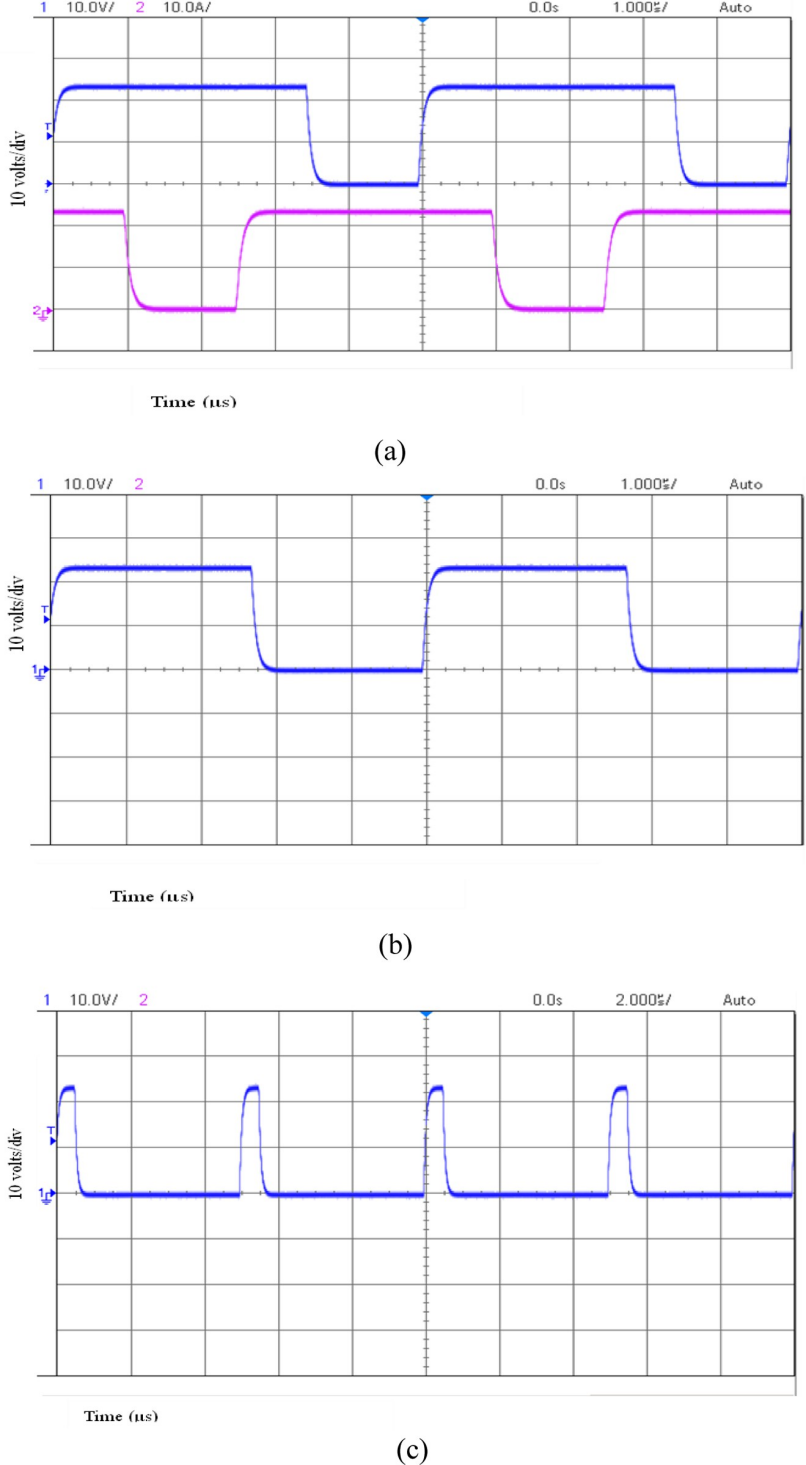
(a) Experimental results of switching pulses *S*_*1*_ and *S*_*2*,_ (b) Experimental results of switching pulses *S*_*3*_ and *S*_*4*,_ (c) Experimental results of switching pulse *S*_*5*_.

**Fig 14:**
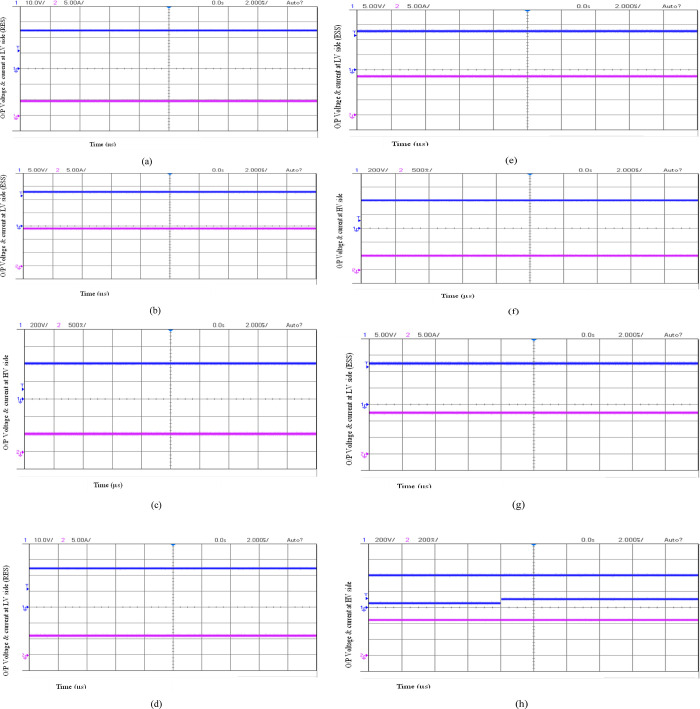
Experimental waveforms for (a) Voltage and current waveforms at RES in Mode 2, (b) Voltage and current waveforms at ESS in Mode 2, (c) Voltage and current waveforms at the DC grid in Mode 2, (d) Voltage and current waveforms at RES in mode 1, (e) Voltage and current waveforms of ESS in mode 1, (f) Voltage and current waveforms at the DC grid in mode 3, (g) Voltage and current waveforms at ESS in mode 3, (h) C. grid voltage for varying voltage at RES.

A 200 W power rating hardware model has been designed, implemented and verified to validate the operation of the proposed converter. The converter circuit was designed for a typical duty ratio of 0:65. The PIC16F877A microcontroller generated switching pulses with a switching frequency of 100 kHz. The gate driver circuit comprises a TLP250 driver IC, and the power circuit is built with MOSFET IRF540. The converter is powered by 24V from a regulated power supply and a 12V battery source. A resistive load is used to evaluate the load. The components details used to build the hardware prototype are shown in Table 3, and the experimental laboratory setup is shown in Fig 12, and the waveform of the result is displayed in Figs 13 and 14.

**Table 3:** Hardware component values.

Item	Component	Rating
Inductor	*L*_*1*_ *-L*_*4*_	100μH
Capacitor	*C*_*1*_ *-C*_*6*_	10μF
Capacitor	*CO*	22μF
MOSFET- IRF540	*S*_*1*_ *-S*_*5*_	100V, 33A
Diode- MBR40250G	*D*_*1*_ *-D*_*13*_	250V, 40A

The gating pulses of *S*_*1*_ *and S*_*2*_ are shown in Fig 13(A) with a duty ratio of 65% used during interleaved boost operation. BS3 gating pulses are demonstrated in Fig 13(B) with a duty ratio of 55% during power transfer from RES to ESS. The gating pulse of *S*_*3*_ is shown in Fig 13(C) with a duty ratio of 11% during power transfer from the DC grid to ESS. These validate the simulation results in Fig 13 for three converter operation modes.

The magnitudes of steady-state voltages and currents at the RES interface, the ESS interface, and the DC grid interface of the converter for mode two operation are shown in Fig 14(A)–14(C), respectively. The resulting steady-state voltages at the RES, EES, and DC grid, which were at nominal values of 24 V, 12 V, and 400 V, validate the accuracy of the duty control of the power switches. The power associated with the RES, EES, and DC grid at similar steady-state values of 60 W, 144 W, and 200 W validate the mode one operation shown in Fig 11. The magnitudes of steady-state voltages and currents at the RES interface ESS interface of the converter are shown in Fig 14(D) and 14(E), respectively, to validate power conversion for mode one operation, which again proved similar to simulation results shown in Fig 11 for power conversion from RES to ESS. The magnitudes of steady-state voltages and currents at the DC grid interface and the ESS interface of the converter are shown in Fig 14(F) and 14(G), respectively, to validate power transfer from the DC grid to ESS about mode three operations with DC grid voltage at 400 V and reversal of power flow into ESS similar to simulation resulted obtained for mode 3. Also, the voltage regulation at the DC grid was verified through experimental results, as shown in Fig 14(H), which shows the stringent voltage regulation with negligible variation in the DC grid for change in RES source voltage, validating the proposed controller’s duty control.

### 4.3 Discussion

The efficiency of the proposed converter in various power transfer modes is obtained as follows:

During power transfer to the DC microgrid, the conduction and switching losses for each of the power switches are described.


$$P_{sws1}=P_{sws2}=\frac{1}{6}*f_{sw}*(t_{on}+t_{off})$$ (39)

$$P_{cs1}={\left(D*i_{RESrms}\right)}^{2}*R_{DSon}$$ (40)

$$P_{cs2}={\left(D*i_{ESSrms}\right)}^{2}*R_{DSon}$$ (41)

Where, *t*_*on*_,*t*_*off*_,*R*_*DSon*_ are the ON, OFF switching times and ON state resistance of power switches, respectively.

With *R*_*Selx*_ being the series parasitic resistance of interleaving inductors, the power loss in interleaving inductors can be obtained as

$$P_{l1}={\left(D*i_{RESrms}\right)}^{2}*R_{Sel1}$$ (42)

$$P_{l2}={\left(D*i_{ESSrms}\right)}^{2}*R_{Sel2}$$ (43)

Total power loss considering these non-idealities in power switches and interleaving inductors,

$$P_{lossf}=P_{sws1}+P_{sws1}+P_{cs1}+P_{cs2}+P_{l1}+P_{l2}$$ (44)

Thus, the efficiency during this mode of operation is high. high. high.


$$\%\eta =\left(1-\frac{P_{lossf}}{\left(V_{RES}*i_{RES})+(V_{ESS}*i_{ESS}\right)}\right)*100$$ (45)

During power transfer from the DC microgrid to the ESS, the conduction and switching losses for each of the power switches are described.


$$P_{sws3}=\frac{1}{6}*f_{sw}*(t_{on}+t_{off})$$ (46)

$$P_{cs3}={\left(\frac{i_{ESSrms}}{D}\right)}^{2}*R_{DSon}$$ (47)

$$P_{cs4}={\left(i_{ESSrms}\right)}^{2}*R_{DSon}$$ (48)

$$P_{l3}={\left(D*i_{RESrms}\right)}^{2}*R_{Sel3}$$ (49)

$$P_{l2}={\left(D*i_{ESSrms}\right)}^{2}*R_{Sel2}$$ (50)

Total power loss, considering these non-idealities in power switches and interleaving inductors,

$$P_{lossr}=P_{sws3}+P_{cs3}+P_{cs4}+P_{l3}+P_{l3}$$ (51)

Thus, the efficiency during this mode of operation is

$$\%\eta =\left(1-\frac{P_{lossr}}{\left(V_{RES}*i_{RES})+(V_{ESS}*i_{ESS}\right)}\right)*100$$ (52)

During the power transfer from RES to ESS, the conduction and switching losses for each of the power switches are described. Owing to the nature of bidirectional switches during this mode of operations the switching and conduction losses are doubled to account for two power switches in bi-directional switch module. Therefore,

$$P_{swbs3}=\frac{1}{3}*f_{sw}*(t_{on}+t_{off})$$ (53)

$$P_{cbs3}={\left(\frac{i_{RESrms}}{D}\right)}^{2}*R_{DSon}$$ (54)

$$P_{cbs1}=2*{\left(i_{RESrms}\right)}^{2}*R_{DSon}$$ (55)

$$P_{cbs2}={2*(i_{ESSrms})}^{2}*R_{DSon}$$ (56)

Total power loss, considering these non-idealities in power switches and interleaving inductors,

$$P_{lossi}=P_{swbs3}+P_{cbs3}+P_{cbs1}+P_{cbs2}$$ (57)

Thus, the efficiency during this mode of operation is

$$\%\eta =\left(1-\frac{P_{losi}}{\left(V_{RES}*i_{RES})+(V_{ESS}*i_{ESS}\right)}\right)*100$$ (58)

The calculated power and efficiency calculations during the different power transfer modes according to Eqs (39)–(58) are listed in Table 4.

**Table 4:** Calculation of power and efficiency.

Duty Ratio	Renewable Energy Sources			Energy Storage System			DC Bus			η
	V	I	P	V	I	P	V	I	P	
0.865	24	3.6	86.4	12	11.5	138	404.2	0.5053	204.24	91.02
0.765	24	9.4	225.6				406.1	0.5076	206.14	91.37
0.897				12	18.6	223.2	403.6	0.5045	203.62	91.23
0.55	24	10.8	259.2	12	20	240				92.59
11.5				12	18.3	219.6	400	0.6	240	91.5

**Fig 15:**
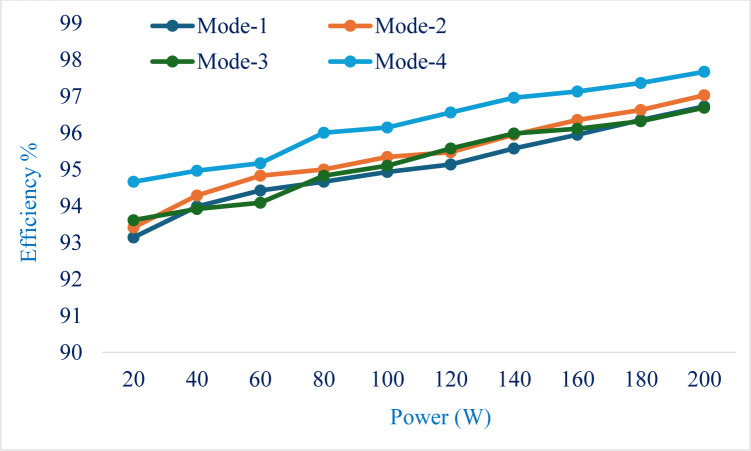
Efficiency against various loading conditions for different modes of power transfer.

During the step-up operation, an efficiency of 91% is obtained, and during the step-down operation, a 92.5% efficiency is obtained. The graph shown in Fig 15 represents the proposed converter’s efficiency under various load conditions for the different power transfer modes. The efficiency is slightly low at 10% of the rated load, and the value increases and reaches 97% of efficiency at the rated load condition of 200W. As the combination of operating switches differs in different operating modes, the efficiency curve also differs in each mode.

Table 5 shows the main performance parameters of 8 high-gain bidirectional converters [22–29] and ten unidirectional converters [30–39] compared to the proposed topology. The proposed converter has higher efficiency and higher voltage gain, and Comparisons of the voltage gain in step-up and step-down modes are presented.

**Table 5:** Comparison between the proposed converter and related bi-directional and unidirectional converters.

	Bidirectional Converters										
Papers	[25]	[26]	[27]		[28]	[29]	[30]	[31]	[32]		
Input Voltage Vin	35–50 V	24–55 V	48 V		24–58 V	24–48 V	40 V	48 V	48 V		
Output Voltage Vo	400 V	400 V	384 V		400 V	360 V	400 V	380 V	400 V		
Step-down mode Voltage Gain $\frac{VL}{VH}$	$\frac{D1*D2*D3}{N}$	$\frac{{\left(1-D\right)}^{2}}{N}$	$\frac{D}{2N+2}$		$\frac{{\left(1-D\right)}^{2}}{\left(N\right)}$	$\frac{D}{2N}$	$\frac{D}{N+1}$	$\frac{D}{1+(1-D)(N+Na)}$	$\frac{D}{2(N+1)}$		
Step-up mode Voltage Gain $\frac{VH}{VL}$	$\frac{N}{\left(D1*D2*D3\right)}$	$\frac{N}{({1-D)}^{2}}$	$\frac{\left(2N+2\right)}{\left(1-D\right)}$		$\frac{N}{({1-D)}^{2}}$	$\frac{1*2N}{\left(1-D\right)}$	$\frac{N+1}{\left(1-D\right)}$	$\frac{1+D(N+Na)}{\left(1-D\right)}$	$\frac{2(N+1)}{\left(1-D\right)}$		
No of Capacitors	3	3	4		4	6	2	1	3		
No of Inductors	2	1	0		1	1	0	0	0		
No of Diodes	0	0	0		0	0	0	0	3		
No of Switches	10	6	6		6	4	4	3	4		
No of Coupled Inductors	1	1	1		1	1	1	1	2		
PWM Control Signals	Complex	Normal	Normal		Normal	Normal	Normal	Normal	complex		
Power output	1000 W	500 W	250 W		1000 W	250 W	300 W	300 W	400 W		
Step-down mode efficiency	94%	94%	96%		95%	93%	94%	95%	95%		
Step-up mode efficiency	94%	96%	96%		96%	94%	94%	94%	95%		
Isolated	Yes	Yes	No		Yes	Yes	No	No	No		
	Unidirectional Converters										
Papers	[33]	[34]	[35]	[36]	[37]	[38]	[39]	[40]	[41]	[42]	Pro*
Input Voltage Vin	40V	24V	20V	12v 24v 36v	24V	24V	16V	24V	48V	12V	24-58V
Output Voltage Vo	200V	380V	410V	310V	156V	156V	160V	160V	500V	60V	400V
Step-down mode Voltage Gain $\frac{VL}{VH}$	----	---	-----	----	---	-----	----	---	-----	----	$\frac{D}{\left(4-D)(1-D\right)}$
Step-up mode Voltage Gain $\frac{VH}{VL}$	$\frac{1}{\left(1-D\right)}$	$\frac{\left(2N+2\right)}{\left(1-D\right)}$	$\frac{N}{\left(1-D\right)}$	$V_{O}=\frac{\left[1+(\alpha_{2}+\alpha_{3})(\alpha_{4}-2)]V_{1}+(2-\alpha_{4})[(\alpha_{1}+\alpha_{2})V_{2}+\alpha_{3}V_{3}\right]}{{\left(1-\alpha_{4}\right)}^{2}}$ (Output voltage)	$\frac{4}{\left(1-D\right)}$	$\frac{4}{\left(1-D\right)}$	$\frac{6}{\left(1-D\right)}$	$\frac{4}{\left(1-D\right)}$	$\frac{3+D}{\left(1-D\right)}$	$\frac{3}{\left(1-D\right)}$	$\frac{\left(2N+1\right)}{\left(1-D\right)}$
No of Capacitors	1	4	7	2	4	4	6	5	3	5	0
No of Inductors	6	0	2	2	2	2	2	2	4	1	4
No of Diodes	3	4	6	4	4	5	7	6	7	5	12
No of Switches	3	2	2	4	1	1	1	1	2	1	10
No of Coupled Inductors	0	2	3	0	0	0	0	0	0	0	0
PWM Control Signals	Normal	Normal	Normal	Normal	Normal	Normal	Normal	Normal	Normal	Normal	Normal
Power output	240W	225W	400W	600W	200W	200W	200W	200W	650W	-----	200W
Step-up mode efficiency	94.8%	91.6%	95.1%	96.4%	-------	96%	95.5%	96%	94%	-----	91%
Step-down mode efficiency	-----	-------	-------	-------	-------	-------	-------	-------	-------	-----	97%
Isolated	No	No	No	No	No	No	No	No	No	No	No

## 5. Conclusions

This paper presented a high voltage ratio non-isolated interleaved bidirectional DC-DC converter consisting of a voltage multiplier circuit, an interleaved boost stage, and a resonant power module. The following benefits are obtained from the proposed converter. A high voltage transfer ratio of 33.33 is available without using a transformer. Also, to get the required high-voltage DC, expansion of the voltage multiplier circuit is allowed. The steady-state results proved the accurate regulation of current at the source ports. Automatic selection among five converter modes to transfer power among three interfaces is formulated, verified with simulation results, and validated through experimental results. The steady-state voltages and currents were stable at nominal values of 24V, 12V, and 400V for the RES, ESS, and DC grid, respectively. Transient waveforms for the DC grid voltage under changing RES conditions proved good voltage regulation at the DC grid with negligible change in grid voltage. The high-voltage gain with a feasible duty range of 54% to 82%) is achieved with the proposed converter. A rigorous comparison is provided with the existing converter, which proved the integrity of the presented topology in terms of gain, efficiency, and operational modes.
